# Scope and magnitude of private sector financing and provision of immunization in Benin, Malawi and Georgia

**DOI:** 10.1016/j.vaccine.2019.05.023

**Published:** 2019-06-12

**Authors:** Ann Levin, Spy Munthali, Venance Vodungbo, Natia Rukhadze, Kuhu Maitra, Tesfaye Ashagari, Logan Brenzel

**Affiliations:** a Levin & Morgan LLC, United States; b University of Malawi, Malawi; c MoH Benin, Benin; d ACT -Global, Georgia; e Abt Associates, United States; f BMGF, United States

**Keywords:** Vaccination, Private sector, Financing, Expenditures

## Abstract

**Background:**

Little is known about the role of private sector providers in providing and financing immunization. To fill this gap, the authors conducted a study in Benin, Malawi, and Georgia to estimate (1) the proportion of vaccinations taking place through the private sector; (2) private expenditures for vaccination; and (3) the extent of regulation.

**Methods:**

In each country, the authors surveyed a stratified random sample of 50 private providers (private for-profit and not-for-profit) using a standardized, pre-tested questionnaire administered by trained enumerators. In addition, the authors conducted 300 or more client exit interviews in each country.

**Results:**

The three countries had different models of private service provision of vaccination. In Malawi, 44% of private facilities, predominantly faith-based organizations, administered an estimated 27% of all vaccinations. In Benin, 18% of private for-profit and not-for-profit facilities provided vaccinations, accounting for 8% of total vaccinations. In Georgia, all sample facilities were privately managed, and conducted 100% of private vaccinations. In all three countries, the Ministries of Health (MoHs) supplied vaccines and other support to private facilities. The study found that 6–76% of clients paid nominal fees for vaccination cards and services, and a small percentage (2–26%) chose to pay higher fees for vaccines not within their countries’ national schedules. The percentage of private expenditure on vaccination was less than 1% of national health expenditures. The case studies revealed that service quality at private facilities was mixed, a finding that is similar to those of other studies on private sector vaccination. The three countries varied in how well the MoHs managed and supervised private sector services.

**Discussion/Conclusion:**

The private sector plays a growing role in lower-income countries and is expanding access to services. Governments’ ability to regulate and monitor immunization services and promote quality and affordable services in the private sector should be a priority.

## 1 Introduction

In low- and middle-income countries (LMICs), national immunization programs (NIPs) manage vaccination service delivery with contributions from the private sector, both not-for-profit and for-profit providers. Governments are the more appropriate source of financing for immunization services, since they make the immunization policies and can ensure that most vaccination services are offered at no charge to the public. Generally, the immunization program is fully or partially financed by the government, sometimes with assistance from external partners. However, access to immunization services is sometimes limited if a country’s macroeconomic status is poor, it lacks health infrastructure, and/or it has competing health priorities. Private for-profit and not-for-profit providers sometimes provide immunization services to fill these gaps and increase access to services. However, little is known about the extent of private provider vaccination in LMICs.

Many studies have examined the role of the private sector in the provision of health services [1], [2], [3], [4]. They have found that governments are not always well equipped and financed to provide preventive health services to their population. The private sector, which includes both for-profit and not-for-profit providers, often provides preventive health services and improves access to these services. However, governmental regulation and supervision of private sector services vary by LMIC [5].

Some studies have researched the role of the private sector in immunization service delivery. A recent study has estimated the proportion of private facilities that provide immunization services using Service Provision Assessments. The study, covering private facilities in Tanzania, Senegal, and Malawi, found that a smaller proportion of for-profit facilities offered child vaccination services (country range, 25–37%) than did public facilities (range, 90–96%) [6]. These data, however, estimated only the number of private facilities that provided vaccination, and not the average number of vaccination services offered through the private sector.

Another study [7], using data from the 1995–96 India National Sample Survey, found that 17% of children and 36% of pregnant women obtained their vaccinations through the private sector. Other studies have estimated the proportion of vaccinations conducted by the private sector through subnational surveys and interviews with key informants [8].

A few studies [9], [10] have focused on whether private providers are offering quality services. They found that immunization service delivery in the private for-profit sector is sometimes associated with poor performance due to lack of training, quality standards, and program monitoring, and to limited supervision from governments.

While the studies on private sector immunization provided insight into the quantity of services provided, as well as the quality of services provided, limited information exists on the role of the private sector in immunization with respect to services and expenditure within LMICs [8].

The World Health Organization has produced a guidance document on the engagement of private providers in immunization service delivery [11]. Recognizing the role that private providers have in immunization, it recommends that the role of private sector providers be assessed in countries so that governments can engage in collaboration and communication with these providers.

To understand the role of private sector provision and financing of vaccination services, the authors conducted three case studies in Benin, Malawi, and Georgia, on the proportion of vaccinations taking place through the private sector, private expenditures for vaccination, and the extent of government support and regulation. This paper synthesizes the findings of the three case studies. Specifically, the paper presents the findings on the role of different private providers in each country with respect to immunization service delivery and financing. The study also contrasts facility official charges for immunization with amounts self-reported by clients for immunization services. In addition, the study compares the quality of services and characteristics of the facilities; highlights the role of the government vis-à-vis private providers; and examines the MoH support and regulation of private sector providers.

## 2 Methods

We selected Benin and Malawi due to their categorization as low-income countries (World Bank 2018 (Table 1) and private sector mapping by Abt’s Strengthening Health Outcomes through the Private Sector (SHOPS) project [12], [13]. The third country, Georgia, was selected due to its categorization as a lower middle-income country, and the privatization of most of its health facilities. It's Social Security Administration contracts all health facilities to provide vaccination services.

**Table 1:** Characteristics of Case Study Countries.

Country (GAVI status)	GNI per capita 2017 in USD (PPP International $, 2017)	Immunization Coverage (Latest Survey)	SHOPS Mapped Private Sector	Location of Private For-Profit Companies, Faith-Based Organizations (FBOs), and Nongovernmental Organizations (NGOs) with Vaccination	Other Considerations
Low-Income					
Benin (GAVI-eligible)	$800 (2260 PPP$)	2013 Survey	Yes	Most facilities in south are PFP*; NGOs and FBOs throughout country; majority in urban areas	18% of total health facilities with vaccination services are private
		BCG: 90%			
		Penta3: 74%			
		MV1: NA			
Malawi (Gavi-eligible)	$320 (1180 PPP$)	2015–16 Survey	Yes	Majority of private facilities offering vaccination are FBOs; more in rural than urban areas	35% of total health facilities with vaccination services are private
		BCG: 98%			
		Penta3: 93%			
		MR: 91%			

Lower-Middle-Income					
Georgia (Graduated from GAVI)	$3780 (10,110 PPP$)	2015–16 Survey	No	PFPs found throughout the country	All facilities contracted to provide vaccination
		BCG: 86%			
		Penta: 88%			
		MMR: 76%			

Source data: World Bank; apps.who.int/immunization_monitoring/global summary; SHOPS project (Abt Associates); MoH Malawi and ICF International 2014.

NGO = nongovernmental organization; FBO = faith-based organization; PFP = Private for-profit; BCG = Bacillus Calmette–Guérin; MCV – Measles; MR = Measles-rubella; MMR = Measles-mumps-rubella.

* More PFPs provide vaccination in Benin due to their greater number, although a larger percentage of NGOs and FBOs than PFPs offer vaccination. PPP = Purchasing Power Parity.

In Benin and Malawi, a stratified random sample of 50 or more private sector providers, drawn from an enumeration conducted by the SHOPS project, was surveyed using a pre-tested, standardized questionnaire (Table 2). The interviewers administered two questionnaires to each private sector provider: a health provider survey and a vaccination client survey. The sample differed in Georgia due to most of its health facilities being private but without being FBOs and NGOs. Thus, the Georgian study team selected all facilities (44) that offered both NIP and non-NIP vaccines (also known as commercial vaccines) in order to obtain data on private expenditures. The study team also collected data at three facilities that provided only non-NIP vaccines, and three facilities that provided only NIP vaccines, for comparison purposes. In all three countries, a total of 300 or more client exit interviews were conducted using a separate pre-tested questionnaire.

**Table 2:** Data collection for three case studies.

	Benin	Malawi	Georgia
Private facilities	35 PFP	16 PFP	44 PFP
	9 FBOs	32 FBOs	3 maternities
	6 NGOs	5 NGOs	3 hospitals
Public facilities	10	11	NA
Vaccination clients interviewed	295	310	301
Dates of data collection	May – June 2017	July 2017	September – October 2017
Location	19 *arrondissements* in 3 southern departments	10 districts in Northern, Southern, and Central Regions	Urban areas of Tbilisi, Kutaisi, and Batumi
Sampling frame	Stratified random sampling	Stratified random sampling	Sampled all facilities w/ commercial vaccination (47) and 3 facilities with only state vaccination

Location			
Urban	82%	38%	100%
Rural	18%	62%	0%

PFP = Private for-profit facilities, FBOs = faith-based organizations, NGOs = non-governmental organizations.

The survey instruments were translated into local languages in each country. Training of interviewers took place during one to two weeks in each country. The health facility survey contained questions on the following topics: (1) list of vaccines provided at the fixed facility and sites; (2) fee structure for vaccination services; (3) support received from the government for commodities, training, and supervision; (4) characteristics of vaccinators; (5) vaccine storage; and (6) availability of vaccines. The exit interview instrument had questions on the characteristics of clients, vaccines used, fees charged, and client satisfaction.

Study teams spent two to three weeks in each country collecting data. After data collection, the data were entered with double entry into Excel files. The study team analyzed the survey data on private sector vaccination using Stata software version 15.1. Statistics were generated on the characteristics of the private sector immunization services, MoH support and monitoring of private sector vaccination, service quality, share of total vaccinations, and share of private expenditures.

The share of total vaccinations provided by private providers in 2017 was calculated for Benin and Malawi, but not in Georgia, given that its proportion was already known (100%). The following methods were used to project the proportion of total vaccinations: (1) estimation of the average number of vaccination services provided through the private sector by type, and multiplying doses provided for each vaccine for each type of private facility by the number of private facilities; and (2) division of private sector vaccinations by the estimate of total vaccinations conducted (coverage for each vaccine multiplied by number of surviving infants). The formula used is summarized below, where Ps represents each private facility by type of facility:

 (Vaccination services_PSi_ X # facilities_Psi_/(vaccination coverage_i_ * surviving infants)

The team also estimated private expenditures on immunization by type and ownership. First, they disaggregated expenditures on vaccinations by type: vaccination cards, service fees, vaccine fees, and syringes. They estimated total expenditures by multiplying average expenditures by the number of facilities by ownership. Second, they divided total private sector expenditures on vaccination by three benchmarks: (1) expenditures on immunization, (2) national private expenditures on health, and (3) national health expenditures.

## 3 Results

Table 3 shows the characteristics of private sector immunization in the three countries. A smaller percentage of private facilities are providing vaccination in Benin (18%) than in the other two countries: 44%-47% and 100% in Malawi and Georgia, respectively. While the majority of private sector vaccination in Benin (77%) and Georgia (100%) occurs in private for-profit facilities and at fixed sites, most private sector vaccination (74%^1^) in Malawi takes place at FBOs and at both fixed and outreach sites.

**Table 3:** Provision of Vaccination in Private Facilities by Country (2017).

	Benin N = 50	Malawi N = 53	Georgia N = 50
Availability of NIP vaccines in private facilities	18% of private facilities administer NIP vaccines (Carmona et al. 2014); majority are private for-profit	44–47% of private facilities (Carmona 2013, MoH Malawi 2014) administer NIP vaccines; majority are Christian Health Association of Malawi Facilities	All private facilities with primary health care provide vaccines.
Outreach provision (% facilities with outreach)			All in fixed sites
Private for-profit	6%	56%	
FBO	0%	88%	
NGO	17%	60%	
Public	50%	100%	

Average Pentavalent Vaccinations per Week			
Private for-profit	15	15	10***
FBO	15	65	NA
NGO	12	15	NA
Public	33	51	NA
Health personnel in private facilities	Private facility vaccinators, primarily nurses	MoH vaccinators (health surveillance assistants), with private personnel support	Consultation by physicians/ vaccination by nurses
Sale of commercial vaccines	Sold in some private and public facilities*	None	Sold in some facilities, mostly in Tbilisi

Vaccine provision at private facilities			
Pentavalent	88%	100%	86%
Measles-containing	70%	100%	88%
Tetanus toxoid only	11%	0%	6%**
Non-NIP vaccines only	4%	0%	6%

NIP = national immunization program.

* Commercial vaccines sold in Benin include MMR, Hepatitis A, Pentaxim, Typhoid, Pneu23, Tetraxim, Euvax, Meningococcal **Commercial vaccines sold in Georgia include Hexavalent (DTP-HepB-Hib-IPV), OPV, PCV, Rotavirus. DT, MMR, IPV, TT, Hepatitis B, DPT, Influenza, Pentaxim, Chickenpox, Tetraxim, Yellow fever, Hepatitis A, Rabies, HPV, Td, Meningococcal.

** Provide a combination of vaccines for pregnant women and newborns: BCG, Hep B, Influenza, Tetanus Toxoid.

*** Hexavalent rather than Pentavalent.

The type of health personnel administering vaccinations in private facilities varied among the three countries. In Benin, nurses employed by private providers administer the vaccinations, while in Malawi, government vaccinators (health surveillance assistants) administer the vaccinations in private facilities with assistance from private sector personnel. In Georgian facilities, doctors conduct consultations with clients and nurses administer the vaccinations.

The provision of non-NIP vaccines (commercial) also varied by country. Private facilities in Malawi only offered NIP vaccines, while many private providers in Benin and Georgia provided both NIP and non-NIP vaccines. In Benin, a few public facilities also sold non-NIP vaccines, due to demand for vaccines for travel purposes, such as to Mecca for the Haj.

The private facilities differ in the vaccines that they offer in the three countries. In Malawi and Georgia, most facilities offered the full range of NIP vaccines. In Benin, 88% of facilities offered vaccines given to infants before six months of age, and 70% of facilities offered vaccines that are given after six months, such as measles-containing ones. In Benin and Georgia, maternities^2^ offer only vaccines required for pregnant women and newborn infants, and a few facilities offer only non-NIP vaccines.

In both Benin and Malawi, the MoH authorizes facilities to provide vaccinations if these facilities are assessed to be qualified and are willing to offer vaccination services. An additional criterion is that nearby public facilities must have a defined need for additional access to vaccination services. In Georgia, if a health facility wants to provide vaccination services, it must notify the State Regulation Agency for Medical Activities and have a vaccination room that meets all of the governmental requirements such as storing vaccines at recommended temperatures.

Table 4 shows indicators of MOH/NIP support to private health facilities. In all three countries, the MoHs supply NIP vaccines, injection supplies, and other supplies to private health facilities. In addition, they supervise the vaccination services in most private facilities. Most personnel (78%-94%) reported receiving government training on introducing new vaccines during the last two years. Fewer personnel among private for-profit facilities reported receiving training on improving vaccination service delivery; personnel reported that 55% − 90% received this training, with the fewest in Malawi and most in Georgia.

**Table 4:** MoH Support to Private Facilities.

Indicator of MoH support to Private Health Facilities	Benin	Malawi	Georgia
MoH supplies vaccines and injection supplies			
Private for-profit	94%	100%	100%
FBO	100%	100%	NA
NGO	100%	100%	NA
Public	100%	100%	

MoH provides cold chain equipment to facilities			
Private for-profit	14%	56%	46%
FBO	44%	88%	NA
NGO	0%	40%	NA
Public	100%	100%	

MoH supervises vaccination services			
Private for-profit	83%	94%	94%/88%*
FBO	100%	94%	NA
NGO	100%	100%	NA
Public	100%	100%	NA

Facility personnel trained in last two years (%) on**:**			
**New Vaccines**			
Private for-profit	77%	94%	98%
FBO	78%	94%	NA
NGO	67%	100%	NA
Public			
	100%	100%	
**Improving Service Delivery**			90%
Private for-profit	77%	50%	NA
FBO	67%	56%	NA
NGO	17%	60%	
Public	90%	36%	
Facilities send monthly reports to government on NIP vaccinations	96%	100%	96%/68*

* Refers to NIP and non-NIP vaccination.

The proportion of private facilities that received MoH cold chain equipment was highest in Malawi and lower in Benin and Georgia. In Malawi, the MoH supplied equipment to most FBO facilities (88%) but to fewer PFPs and NGOs (56% and 40%, respectively). In Benin and Georgia, MoHs supplied cold chain equipment to fewer than 50% of private health facilities.

In the three countries, most health facilities send monthly reports on their immunization service provision of NIP vaccines to the government. However, fewer Georgian facilities reported on the provision of commercial vaccination to the government.

Table 5 shows the findings on out-of-pocket or private expenditures on vaccination in private facilities. In Benin, about three-quarters of facilities charge for vaccination cards, a third charge for vaccination services, and less than a tenth charge for vaccines. In Malawi, on the other hand, there are relatively fewer fees – 6% for vaccination cards, 2% for services, and 2% for vaccines. In Georgia, slightly more than a third of the facilities charge for registration when the client is not registered at the facility. Less than a fifth (16%) charge for consultations, but most charge for non-NIP vaccines. Only a small percentage of clients, 4%, 8%, and 15%, respectively, have private insurance or prepaid plans and most of these charges are not reimbursable (e.g. only 15% of clients with private insurance in Georgia reported that the fees were reimbursable).

**Table 5:** Facility Charges for Vaccination and Median Private Expenditures for Immunization, 2017 (U.S. Dollars).

	Benin	Malawi	Georgia
Percentage of private facilities charging as per facility interviews			
Card	76%	6%	NA
Registration	NA	NA	36%*
Consultation	NA	NA	16%
Vaccination Service	34%	2%	0
Vaccine	8%	2%	8%**/94%***
Percentage of clients reporting paying for vaccination during exit interviews	64%	14%	19%

Client reported paying for:			
Card	35%	20%	NA
Registration	NA	NA	2%
Consultation	NA	NA	11%
Vaccination service	30%	13%	0
Vaccine	3%	8%	26%
How much paid according to client exit interviews – median (mean in parentheses)			
Card	$0.17 ($0.52)	$0.28 ($0.28)	NA
Registration	NA	NA	$3.90 ($4.39)
Consultation	NA	NA	$9.77 ($10.90)
Vaccination Service	$0.35 ($0.54)	$0.21 ($0.64)	0
Vaccine	$13.09 ($9.57)	$0.48 ($0.46)	$13.67 ($22.66)

Private insurance or prepaid plan	4%	8%	15%****

* For persons not registered.

** For NIP vaccines.

*** For non-NIP/commercial vaccines Note: NA is used when item or service not in program.

**** 15% of clients with insurance reported that vaccination fees were reimbursable.

The percentage of clients that reported paying for vaccination ranged from 14% in Malawi to 64% in Benin. In Benin, 35% paid for cards, 30% for vaccination services, and 3% for vaccines; in Malawi, 20% paid for cards (health passports), 13% for vaccination services, and 8% for vaccines. In Georgia, 2% paid for registration, 11% for consultations, and 26% for vaccines.

In Georgia, some fees are charged only to clients with private insurance, since they can get reimbursed for the services.

### 3.1 Private provider service quality

Table 6 presents indicators of quality for vaccination service delivery in private facilities: accreditation^3^, frequency of regulation and supervisory visits, cold chain equipment, and vaccine availability. Most private facilities were accredited by regulatory bodies in their countries. There was more variation in the frequency of regulatory visits and supervision. The most frequent regulatory visits were reported in Benin (84% in last year) and the least in Georgia (42% in last year). Health providers reported they were supervised most frequently in Malawi (78% monthly or quarterly) and the least often in Benin (54% monthly or quarterly).

**Table 6:** Indicators of Quality in Private Facilities.

Indicators of Quality	Benin	Malawi	Georgia
Accreditation	96%	100%	98%

Last regulatory visit			
<6 months	60%	23%	12%
6–12 months	24%	44%	30%
>12 months	10%	25%	20%
Never	2%	0%	16%
Don’t know	4%	8%	18%

Frequency of supervision			
Monthly	10%	28%	36%
Quarterly	22%	50%	12%
Every 6 months	14%	11%	14%
Every 4 months	22%	NA	14%
Annually	10%	11%	10%

Cold chain			
Stores vaccines*	60% (50–89%)	96% (80–100%)	98%
Equipment meets regulations**	83% (67%-84%)	71% (51–100%)	80%

* Percentage of facilities that have vaccine in stock.

** Percentage of facilities with refrigerators either pre-qualified by WHO or not classified as domestic.

The majority of private facilities reported that they stored vaccines, ranging from 60% in Benin to 98% in Georgia. Among the private facilities that stored vaccines, most had cold-chain equipment that met the national standards (i.e., equipment was either a WHO pre-qualified brand or was a brand approved by the MoH). The percentage of facilities that did not meet standards was lowest in Benin (17%) and highest in Malawi (29%).

Table 7 shows indicators of client satisfaction among the three countries. In terms of information provision by providers, 88% of the clients in Malawi, 90% in Benin, and 97% in Georgia indicated that health workers/vaccinators answered their vaccination queries during their visits. While this is a positive indication, some of the clients believed the information provided was not adequate. Thirteen percent of the private sector clients in Benin and fifteen percent in Malawi were dissatisfied with the amount of explanation provided regarding the immunization service.

**Table 7:** Indicators of Client Satisfaction with Immunization Services in Private Facilities.

Indicators of Quality	Benin	Malawi	Georgia
Client responded that health workers responded to their questions			
Yes	90% (78%)	88% (87%)	97%
No	7% (20%)	12% (7%)	1%
Don’t Know	1% (2%)	0% (7%)	2%

Waiting time median (mean)			
PFP	20 (32)	5 (30)	0 (4.9)
FBO	35 (61)	10 (21)	NA
NGO	38 (43)	13 (37)	NA
Public	38 (65)	20 (34)	

% dissatisfied w/ facility services			
Waiting time	20% (49%)	17% (31%)	2%
Possibility of discussing problems	6% (22%)	12% (12%)	0%
Amount of explanation	13% (22%)	15% (13%)	0%
Availability of vaccines	3% (4%)	7% (4%)	0%
Days that service is available	7% (0%)	7% (5%)	1%
How well treated	1% (4%)	3% (12%)	1%
Cost of services	8% (10%)	7% (2%)	1%

If not at the facility closest to your home, why not?			
Hours of service	6% (2%)	3% (4%)	2%
Bad reputation	5% (2%)	3% (0%)	2%
Don’t like facility	4% (0%)	3% (0%)	2%
Poor availability of vaccines	3% (2%)	1% (2%)	1%
High cost	8% (0%)	1% (3%)	0%
Worker attitude	16% (6%)	0% (0%)	0%

Note: Public facility percentages are shown in percentages.

While the waiting time for service was equal to or lower than that in public facilities, clients still responded that they were most dissatisfied with the waiting time in the facilities. Waiting time for services was longest in Benin, where the median time ranged from 20 to 38 min; in Malawi, the median waiting time was 5–13 min; and in Georgia, the waiting time was the lowest, with a median time of less than a minute and a mean of five minutes.

Only 1–8% of clients indicated that they were dissatisfied with the cost of the service. Clients who had traveled further from their home to other private health facilities did so for the following reasons: in Benin, they preferred the worker attitude and lower cost at their health facility relative to the closer facility; in Malawi and Georgia, clients preferred the hours of service and reputation at the health facility compared to the one at the closer facility.

### 3.2 Proportion of vaccination services that are private

The estimated proportion of vaccination services administered through private providers was 7.8% in Benin, 26.6% in Malawi, and 100% in Georgia (Table 8).

**Table 8:** Proportion of Vaccinations That are Private during 2017.

	Benin	Malawi	Georgia
National program vaccines given in the sample of private facilities	269,742	2,208,000	354,330
Non-NIP vaccines given in the sample of private facilities	10,097	–	23,400
Total private vaccinations in the sample of private facilities	279,839	2,208,000	36,407
Projected number of annual vaccinations given in the private sector for the country*	2,579,934	8,314,410	354,300
Estimated percentage of total annual vaccinations given in the private sector	7.8%	26.6%	100%

* Target population multiplied by survey coverage rates for each vaccine.

### 3.3 Total private expenditures estimate

Table 9 shows the total estimated 2017 private expenditures spent on vaccination. The total private spending on immunization ranged from $124,000 in Malawi to $716,400 in Benin, and $2.4 million in Georgia. For Benin, the private vaccination expenditures were 5.7% of total immunization expenditures, 0.18% of private health expenditures, and 0.07% of total spending on health. For Malawi, the figure was 0.8% of immunization spending, 0.2% of private health spending, and 0.02% of national health spending. For Georgia, it was 13.2% of immunization spending, 6.5% of private health expenditures, and 13.2% of national health spending.

**Table 9:** Estimation of Private Expenditures on Vaccination (2017).

	Benin (thousands)	Malawi (thousands)	Georgia (millions)
Private expenditures on card/service/vaccine in sampled facilities	$716.4	$124.4	$2.4
Total private spending on health (data source?)	$398,800	$65,788	$14.7
% private expenditures for immunization	0.18%	0.2%	6.5%
Total national spending on health	$1,100,000	$707,400	$1,127
% of national health spending that is private expenditures for immunization	0.07%	0.02%	0.08%
WHO-UNICEF Joint Reporting Form estimate of total spending on immunization	$12,700	$16,200	$7.1
% of total spending on immunization that is private expenditures for immunization	5.7%	0.8%	13.2%

## 4 Discussion

In the three study countries, private providers are playing an important role in increasing access to immunization, including non-NIP vaccines, but the models of public-private engagement in vaccination services vary widely. Specifically, the proportion of private facilities offering vaccination differs among the three countries. First, in Benin, slightly fewer than 20% of private health facilities offer vaccination, the majority of which are private for-profit (although a lower percentage of private for-profit facilities offer vaccination than do FBOs and NGOs). In Malawi, public vaccinators administer NIP vaccines in approximately 40% of private health facilities, mostly FBOs. In Georgia, the Social Security Administration contracts all health facilities (100%) to provide vaccination.

When the contribution to vaccination coverage by the private sector is estimated taking into account average service volume, it ranges from 8% to 100%. The percentage is low in Benin (8%), because average vaccination service volume at private facilities is relatively lower than it is in public facilities, and the proportion of private facilities providing this service is small. In Malawi, the service volume and proportion of private facilities offering this service is approximately twice as high as in Benin, and the proportion of total vaccinations that are private is 27%. Finally, in contrast, in Georgia, all vaccination services are considered private since the services are contracted by the government.

The level of public-private engagement in vaccination is likely also affected by whether the country is GAVI eligible since countries that are graduating or have graduated will be more interested in the financial sustainability of their program. Georgia, the country that has graduated from GAVI, has the most public-private engagement in vaccination.

This study adds to the current literature by evaluating not only the proportion of private providers offering vaccination in low- and middle-income countries, but also the proportion of total annual vaccination that is private. Study methods took into account average vaccination service volume at private facilities, as well as the proportion of facilities that are private. Only a few other studies have estimated the proportion of vaccinations offered through the private sector (e.g., [15], [8], [16]).

This study was also able to evaluate the proportion of clients that paid fees for vaccination, ranging from 14% in Malawi to 64% in Benin. Total private expenditures for immunization in comparison to total private health expenditures and total health spending are less than 1% in all of the countries, and are relatively low.

The fees are low for vaccination cards and for services for provision of NIP vaccines, but are higher for non-NIP vaccines in Benin and Georgia and consultations in Georgia. Fees for vaccination seem unlikely to affect access to and use of vaccines in general, since the higher fees are usually for clients wishing to purchase non-NIP vaccines. However, low-income clients may find that fees for vaccination cards and services could be a deterrent to use of services.

The case studies revealed that service quality at private facilities was mixed. While all countries had regulatory and supervisory visits at private facilities, training, regulatory and supervisory visits were sometimes infrequent, some facilities had cold chain equipment that does not meet national regulations, and waiting time at facilities was a source of dissatisfaction among clients^4^. These findings are in alignment with results of other studies [9], [10]. In addition, Olorunsaiye at al. [6] found that longer waiting times could be a barrier to vaccination. The private providers reported that most were sending monthly reports on the number and type of NIP vaccinations that they were providing. However, in Benin and Georgia, where there is non-NIP vaccination, there was limited reporting on non-NIP service volume and supervision of this vaccination service. Public facilities were also able to administer more vaccinations per week than the private facilities with the exception of faith-based facilities in Malawi that administered more vaccination per week than the public facilities.

This study also found that in Benin, but not in Malawi and Georgia, private providers were less likely to offer vaccinations that are given to infants after they reach six months, such as measles vaccine and yellow fever. Other studies [18] have similarly found that private facilities sometimes have limited availability of vaccines such as measles.

Private providers in two of the three countries (Benin and Georgia) are offering non-NIP vaccines. The provision of non-NIP vaccines can improve the population’s health if providers are offering vaccines not found in national programs such as influenza. The availability of these vaccines adds to the choice that parents have for their children and themselves. For example, in Georgia, many clients were purchasing influenza vaccine, a vaccine provided within the NIP to special populations only. In addition, the sale of non-NIP vaccines is an important service for people who plan to travel to other countries.

While there are advantages to provision of non-NIP vaccines, the government monitoring of non-NIP vaccines was limited in Benin and Georgia. The private providers reported that they did not send non-NIP vaccination service data to the government. In addition, there is a potential for private providers to create demand for vaccines that are duplicative and costly.

In all three countries, the government has significant engagement with the private sector since they provide vaccines, injection supplies, and other materials as well as training and supervision to the providers. However, in Benin and Malawi, the engagement was closer with faith-based health providers rather than private for-profit and NGO facilities.

**Fig. 1:**
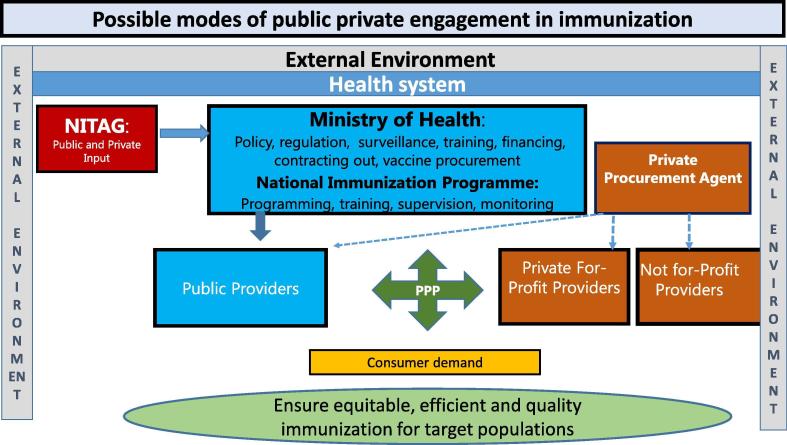
Analytical Framework of Potential Private-Public Partnerships.

Source: [17]

Public-private partnerships in immunization, similar to other programs in the health sector, happen not only at the service provision level but also above facility level around policy and procurement. Fig. 1 shows an analytical framework of possible modes of public-private engagement in immunization. It shows that MoHs oversee NIPs and set policy and norms for vaccination. They also procure vaccines and take advice from evidentiary bodies, such as National Immunization Technical Advisory Groups, on immunization and vaccine policy. Both public facilities and private providers offer immunization services to target populations – children, pregnant women, adolescents, adults, and special populations.

While the government is responsible for setting policy and norms for vaccination, it can improve public-private engagement by involving private sector providers in decision-making on policies that affect the vaccination program. For example, private sector providers could be invited to participate in discussions of vaccination policy at NITAGs, pediatric association meetings, or during training sessions.

Governments can further engage in monitoring the quality of private sector service provision by requiring annual licensing or at least some type of monitoring of quality metrics that is tied to government provision of vaccines. Malawi already has an informal method of ensuring service quality since district program managers decide where vaccinations can be conducted and the government employs government vaccinators. Georgia also has contracts with health facilities to provide primary health care services. Employing regulation strategies such as annual licensing and supervision are a systematic approach to ensuring that quality metrics are maintained in private facilities.

Other potential policy implications for the private sector role in immunization fall under the role of Ministries of Health: regulation, oversight, training, and supervision. All three countries had regulations against charging for vaccines. However, private facilities are charging some fees ed for provision of vaccination services and consultations (not for NIP vaccines) in private facilities. Although private expenditures on immunization were relatively low when compared to total private expenditures on health and total health spending, it is important to monitor these expenditures to ensure that these are not a deterrent to utilization of vaccination services.

To ensure that private providers are offering quality services, governments should guarantee adequate training on improving vaccination service delivery in private facilities. It is particularly important that they provide clear guidance on how to purchase appropriate cold chain equipment for vaccine storage, and information on how to maintain the cold chain. To the extent possible, NIPs should also frequently supervise the private facilities that provide vaccination services, to ensure high-quality services.

Governments also need to improve their monitoring and supervision of provision of non-NIP vaccines. It is important that governments improve their supervision of non-NIP vaccination as well as require that private providers report on their non-NIP service volume. They should ensure that non-NIP vaccines are given to appropriate target populations and are not duplicative.

## Author contributions

AL and LB designed the study. SM, VV, and NR obtained and interpreted the data findings while AL analyzed the data. AL, KM, TA, and LB contributed to the drafting and editing of the manuscript. All authors approved the final submitted version.

## Funding

The authors acknowledge support for this work under Investment ID OPP1129338 to Abt Associates, Inc. by the Bill and Melinda Gates Foundation.

## Declaration of Competing Interest

None.

## Footnotes

1 The proportion of private providers that offer vaccination that are FBOs is 64% if including private company clinics that only offer services to their employees and 74% without company clinics (i.e., only private for-profit, FBO, and NGO) [14].

2 Maternities are either standalone clinics or are wards that are part of larger health facilities.

3 Accreditation is a process by which national regulatory bodies assess the capacity and quality standards of private health facilities and authorize these to provide services.

4 It should be noted, however, that waiting times were longer at public than private facilities.
