# Supplementary material for: Scope and magnitude of private sector financing and provision of immunization in Benin, Malawi and Georgia
Source: Vaccine. 2019 Jun 12;37(27):3568–75. doi: 10.1016/j.vaccine.2019.05.023 (PMC6573791; doi:10.1016/j.vaccine.2019.05.023)
Supplement: Supplementary data 1 [file mmc1.docx]

Facility Survey

Form Number (Unique ID): ___________________

# Preliminary section: Q1-Q7 To be completed before beginning interview

| **S/N** | **Variable** | **Category** | **Code** |
| --- | --- | --- | --- |
|  | Interviewer name |  |  |
|  | Interviewer code |  |  |
|  | Interview date | Day: □□ Month: □□ Year: □□□□ |  |
|  | District (or commune) name |  |  |
|  | Department or Region |  | 1 |
|  | Health Area |  | 1 |
|  | Is the facility located in a rural, urban or peri-urban area? | Rural……………………………………………………………………………………………………………  Peri-urban…………………………………………………………………………………………………  Urban…………………………………………………………………………………………………………. | 1  2  3 |

##

## GPS Coordinates (hddd.ddddd)

| ID | Attitude (metres) |  |  |  |  | . |  |  |  |  |
| --- | --- | --- | --- | --- | --- | --- | --- | --- | --- | --- |
|  | Latitude South |  |  |  | . |  |  |  |  |  |
|  |  | h | d | d | . | d | d | d | d | d |
|  | Longitude East |  |  |  | . |  |  |  |  |  |

## SUPERVISORY REVIEW

| Name of Reviewer | Position of Reviewer | Type of Review | Date of Review | Comments |
| --- | --- | --- | --- | --- |
|  |  |  |  |  |
|  |  |  |  |  |
|  |  |  |  |  |
|  |  |  |  |  |

# Introduction and Informed Consent

Hello, my name is ____. We are here on behalf of _________ to assist the government and donors such as the Gates Foundation to know more about the services provided by private health providers in _______.

Your participation in this study is voluntary. It is up to you to decide whether or not to take part in this study. If you decide to take part in this study, you are still free to withdraw at any time and without giving a reason. You are free to not to answer any question or questions if you choose.

If you choose to participate in this study, information on the location of the facility, the types of services provided, the fees charged for immunization, and the number and type of providers and health workers who work at the facility will be shared. All other information will be kept confidential.

Do you have any questions about the survey at this time?

Do you agree to participate in the survey?

___________________________

Signature of interviewer indicating informed consent was provided

_______________

Date

# Section A – Basic Questions

| **Number** | **Question** | **Answer** | | **Code** |
| --- | --- | --- | --- | --- |
|  | Interview start time  (Use 24 hour time) | hh.mm | . | |
| 1. . | Name of person responding to the survey |  | |  |
|  | Interviewer: note sex of respondent here. | Male……………………………………………………………..……..…….  Female……………………………………………………………….……… | | 1  2 |
| 1. . | Title/position of person responding to the survey *(to facilitate follow-up if needed)* |  | |  |
|  | What is the cadre or qualification of the manager of this facility? | Clinical Officer  Nurse (specify type)……………………………………………….  Physician……………………………………………………………….  Other (specify)………………………………………………………. | | 1  2  3 |
| 1. . | Facility name |  | |  |
|  | Address of facility |  | |  |
|  | Facility phone number |  | |  |
|  | Facility email address |  | |  |
|  | Type of Facility | Private for Profit Health Clinic  Maternity  NGO Clinic  Hospital  Faith-based  Other (specify)…………………………………………………………… | | 1  2  3  4  5  -888 |
|  | Does the facility have access to piped water? | Yes……………………………………………………………………………  No………………………………………………………………………………. | | 1  2 |
|  | Does the facility have reliable 24 hour electricity? | Yes……………………………………………………………………………  No………………………………………………………………………………. | | 1  2 |
|  | Facility operating times (use 24 hour clock)  Probe for the official operating times. | Monday: ___________ to _____________  Tuesday: ___________ to _____________  Wednesday: ___________ to _____________  Thursday: ___________ to _____________  Friday: ___________ to _____________  Saturday: ___________ to _____________  Sunday: ___________ to _____________ | |  |
|  | Is this facility affiliated with any association or network or franchise? | Yes………………………………………………………………………………  No………………………………………… (Skip to Q 23)……..,……. | | 1  2 |
|  | What organizations is this facility affiliated with?  *(Interviewer: Read each option and circle all that apply. Multiple responses allowed.)* |  | | 1  2  3  4  5  6  -888 |
|  | Is your facility registered to accept private medical insurance schemes clients? Community insurance schemes? | Yes………………………………………………………………………………  No…………………………………………………(Skip to Q 25) | | 1  2 |
|  | If yes, from which private medical schemes? | (Insert names of private medical schemes)  Other (specify) ________________________________ | | 1  2  3  -888 |
|  | Do you provide the following maternal and child health (MCH) services at this facility?  END INTERVIEW IF THEY DON’T VACCINATE? OR CONTINUE IF VACCINATION COULD BE OFFERED?  *(Interviewer: read all options and circle all that apply. Multiple responses allowed.)* | Antenatal care (ANC)……………………………………………….  Labor and delivery …………………………………………………….  Routine Immunizations ………………………………..  Sick child treatment ………………………………………………….  Growth monitoring ……………………………………………………  Other (specify) _______________________________  Don't know ………………………………………………………………. | | 1  2  3  4  5  -888  -999 |
|  | \| Now I would like to ask you specifically about vaccination services for children under 5 years. For each of the following services, please tell me whether the routine service is offered by your facility, and if so, *how many days* per month the service is provided at the facility, and how many days per month \| \| \| \| \| \| \| \| \| \| \| \| \| \| \| \| \| \| \| \| \| \| \| \| \| \| \| \| \| \| \| \| \| \| \| \| \| \| \|  \| \| --- \| --- \| --- \| --- \| --- \| --- \| --- \| --- \| --- \| --- \| --- \| --- \| --- \| --- \| --- \| --- \| --- \| --- \| --- \| --- \| --- \| --- \| --- \| --- \| --- \| --- \| --- \| --- \| --- \| --- \| --- \| --- \| --- \| --- \| --- \| --- \| --- \| --- \| --- \| --- \| \| as outreach, if any. \| \| \| \| \| \| \| \| \| \| \| \| \| \| \| \| \| \| \| \| \| \| \| \| \| \| \| \| \| \| \| \| \| \| \| \| \|  \|  \|  \| \|  \|  \|  \|  \|  \|  \|  \|  \|  \|  \|  \|  \|  \|  \|  \|  \|  \|  \|  \|  \|  \|  \|  \|  \|  \|  \|  \|  \|  \|  \|  \|  \|  \|  \|  \|  \|  \|  \|  \|  \| | | |  |
|  | BCG | # of days per month service is provided at Facility  # of days per month service is provided through outreach  Don’t Know…………………………………………………………. | | ____  ____  -999 |
|  | DT | # of days per month service is provided at Facility  # of days per month service is provided through outreach  Don’t Know…………………………………………………………. | | ____  ____  -999 |
|  | DTP-Hib-HepB vaccination (i.e. pentavalent) | # of days per month service is provided at Facility  # of days per month service is provided through outreach  Don’t Know…………………………………………………………. | | ____  ____  -999 |
|  | Oral Polio Vaccine | # of days per month service is provided at Facility  # of days per month service is provided through outreach  Don’t Know…………………………………………………………. | | ____  ____  -999 |
|  | Inactivated Polio Vaccine | # of days per month service is provided at Facility  # of days per month service is provided through outreach  Don’t Know…………………………………………………………. | | ____  ____  -999 |
|  | Measles (or MR or MMR) Vaccination | # of days per month service is provided at Facility  # of days per month service is provided through outreach  Don’t Know…………………………………………………………. | | ____  ____  -999 |
|  | Pneumococcal Vaccine | # of days per month service is provided at Facility  # of days per month service is provided through outreach  Don’t Know…………………………………………………………. | | ____  ____  -999 |
|  | Rotavirus Vaccine | # of days per month service is provided at Facility  # of days per month service is provided through outreach  Don’t Know…………………………………………………………. | | ____  ____  -999 |
| 1. Yell | Yellow fever | # of days per month service is provided at Facility  # of days per month service is provided through outreach  Don’t Know…………………………………………………………. | | ____  ____  -999 |
|  | Other vaccine (specify) | # of days per month service is provided at Facility  # of days per month service is provided through outreach  Don’t Know…………………………………………………………. | | ____  ____  -999 |
|  | Other vaccine (specify) | # of days per month service is provided at Facility  # of days per month service is provided through outreach  Don’t Know………………………………………………………….  Don’t Know………………………………………………………. | | ____  ____  -999 |
|  | On average, how many immunizations in this clinic are given per day by type?  *(Interviewer: Probe if necessary. Use actual records if possible; otherwise ask for recall.)* | BCG  DTP or Pentavalent  Measles  Measles-Rubella  Measles-Rubella-Mumps  OPV  IPV  MONOVALENT Hepatitis B  YF  Pneumococcal  Rotavirus  Other (specify) _______________________________  Don’t know ……………………………………………………………… | | ___  ___  ___  ___  ___  ___  ___  ____  ____  ____  ____  ____ |
|  | On average, how many immunizations are given per outreach session by type?  *(Interviewer: Probe if necessary.* ***Use actual records*** *if possible; otherwise ask for recall.)* | BCG  DTP or Pentavalent  Measles  Measles-Rubella  Measles-Rubella-Mumps  OPV  IPV  Monovalent Hepatitis B  Pneumococcal  Rotavirus  Other (specify) _______________________________  Don’t know ……………………………………………………………… | |  |
|  | \| Now I would like to ask you specifically about vaccination services for pregnant women. For each of the following services, please tell me whether the service is offered by your facility, and if so, *how many days* per month the service is provided at the facility, and how many days per month \| \| \| \| \| \| \| \| \| \| \| \| \| \| \| \| \| \| \| \| \| \| \| \| \| \| \| \| \| --- \| --- \| --- \| --- \| --- \| --- \| --- \| --- \| --- \| --- \| --- \| --- \| --- \| --- \| --- \| --- \| --- \| --- \| --- \| --- \| --- \| --- \| --- \| --- \| --- \| --- \| --- \| --- \| \| as outreach, if any. \| \| \| \| \| \| \| \| \| \| \| \| \| \| \| \| \| \| \| \| \| \| \| \| \| \| \| \|  \|  \|  \|  \|  \|  \|  \|  \|  \|  \|  \|  \|  \|  \|  \|  \|  \|  \|  \|  \|  \|  \|  \|  \|  \|  \| | | |  |
|  | Tetanus Toxoid (TT)/TETANUS DIPHTHERIA (Td or DT)  *(Interviewer: read all options and circle all that apply. Multiple responses allowed.)* | # of days per month service is provided at Facility  # of days per month service is provided through outreach……………………………………………………..  Not provided at this facility………………………….  Don’t Know…………………………………………………………. | | ____  ____  ____  -999 |
|  | Influenza Vaccine | # of days per month service is provided at Facility  # of days per month service is provided through outreach  Not provided at this facility………………………….  Don’t Know…………………………………………………………. | | ____  ____  ____  -999 |
|  | Other vaccine (specify) | # of days per month service is provided at Facility  # of days per month service is provided through outreach ……………………………………………………...  Not applicable………………………………………………  Don’t Know…………………………………………………………. | | ____  ____  -888  -999 |
|  | On average, how many immunizations in this clinic are given to pregnant women per day by type?  *(Interviewer: Probe if necessary. Use actual records if possible; otherwise ask for recall.)* | Tetanus Toxoid (TT)/Td……………………………………………………  Influenza Vaccine……………………………………………….  Other (specify) ___________________________  Don’t know ………………………………………………………. | | ___  ___  ___  ____ |
|  | On average, how many immunizations are given to pregnant women during an *outreach* session by type?  *(Interviewer: Probe if necessary. Use actual records if possible; otherwise ask for recall.)* | Tetanus Toxoid (TT/Td……………………………………………………  Influenza Vaccine…………………………………………………  Other (specify) _____________________________  Don’t know …………………………………………………… | | ___  ___  ___  ____ |
|  | \| Now I would like to ask you specifically about vaccination services for adolescent/pre-adolescent girls). For each of the following services, please tell me whether the service is offered by your facility, and if so, how many days per month the service is provided at the facility, and how many days per month as outreach, if any. \| \| \| --- \| --- \| \|  \| | | |  |
|  | HPV vaccine | # of days per month service is provided at Facility  # of days per month service is provided through outreach  Don’t Know…………………………………………………………. | | _____  ____  -999 |
|  | Other vaccine (specify) | # of days per month service is provided at Facility  # of days per month service is provided through outreach  Don’t Know…………………………………………………………. | | _____  ____  -999 |
|  | On average, how many immunizations in this clinic are given to adolescent girls in a day by type?  *(Interviewer: Probe if necessary. Use actual records if possible; otherwise ask for recall.)* | HPV  Other (specify) _______________________________  Don’t know ……………………………………………………………… | | ___  ___  ___  ___ |
|  | On average, how many immunizations are given to adolescent girls in a day during outreach session by type?  *(Interviewer: Probe if necessary. Use actual records YES if possible; otherwise ask for recall.)* | HPV  Other (specify) _______________________________  Don’t know ……………………………………………………………… | | ___  ___  ___  ___ |
|  | Are any other vaccines offered in your facility? | Other vaccines (specify)……………………………………….  Population getting this vaccine…………………………… | | ____  ____ |
|  | \| Now I would like to ask you specifically about fees charged for vaccination services. \| \| \| --- \| --- \| \|  \| | | |  |
|  | Do clients pay fees for services at this facility? | Yes …………………………………………………………………  No …………. (skip to Q ?)  Only registration…………………………………………….  Other (specify)__________________________  Don’t know……………………………………………………. | | 1  2  3  -888  -999 |
|  | Are the fees at this facility displayed? | Yes …………………………………………………………………  No …………. (skip to Q ?)  Only registration…………………………………………….  Other (specify)__________________________  Don’t know……………………………………………………. | | 1  2  3  -888  -999 |
|  | Do clients pay fees for vaccination? | Yes …………………………………………………………………  No …………. (skip to Q ? )  Only registration…………………………………………….  Other (specify)__________________________  Don’t know……………………………………………………. | | 1  2  3  -888  -999 |
|  | How much do clients pay per vaccination?  WHAT DOES FEE INCLUDE? | BCG………………………………………………………….…………  DTP or Pentavalent.……………………………………………  Measles………………………………………………………………  Measles-Rubella.…………………………………………………  Measles-Rubella-Mumps……………………………………  OPV……………………………………………………………………  IPV……………………………………………………………………..  Monovalent Hepatitis B…………………………………………………………  Pneumococcal…………………………………………………..  Rotavirus…………………………………………………………..  Other (specify) ___________________________  Don’t know YF, HPV, CHICKENPOX, TT/Td/DT……………………………………………………… | | ___  ___  ___  ___  ___  ___  ___  ____  ____  ____  ____  ____ |
|  | Are fees for vaccination covered by any medical scheme/insurance plan? | Yes………………………………………………………………………….  No………………………………………………………………………….  Don’t know  If yes, which schemes  What percentage of costs of fees are covered? (percentage) | | ____  ____  ____  ____  ____ |
|  | Are the fees applicable to all clients? Do some clients receive exemptions from fees? | Yes (skip to Q )  No  Don’t know | | 1  2  -999 |
|  | If there are exemptions from fees, who gets these? | _____________________________ | | ___ |
|  | Now I would like to ask you specifically about your health personnel. | | |  |
|  | What cadre of health worker is administering vaccinations? | Cadre HW providing infant vaccinations  Cadre HW providing ANC vaccinations | | _____  _____  _____ |
|  | How many health workers are generally available to provide vaccination? | # HWs giving Infant vaccinations  # HWs giving ANC vaccinations  # HWs giving adolescent girl’s vaccinations | | _____  _____  _____ |
|  | Has at least one staff member who provides vaccination services at the facility been trained in the last 2 years on using new vaccines?  (If no, skip to Q62) | Yes ………..………………………………………………………………  No …………………………………………………………………………  Don’t know………………………………………………………………… | | 1  2  -999 |
|  | If yes, how many have been trained?  Who conducted the training? | Number trained in giving new vaccines in last two years  Facility In-charge …………………………………………………………..  Headquarters……………………………………………………………  Ministry of Health…………………………………………………….  Other……………………………………………………………………….  Don’t know…………………………………………………………….. | | _____  1  2  3  -888  -999 |
|  | Have any staff received training on improving vaccine service delivery (not new vaccines) in the last two years? | Yes ………..………………………………………………………………  No …………………………………………………………………………  Don’t know………………………………………………………………… | | 1  2  -999 |
|  | 1. If yes, how many have been trained? 2. Who conducted the training? | Number trained in vaccination service delivery in last two years  Facility In-charge…………………………………………………………..  Headquarters……………………………………………………………  Ministry of Health……………………………………………………..  Other………………………………………………………………………..  Don’t know……………………………………………………………… | | _____  1  2  3  -888  -999 |
|  | Now I would like to ask you specifically about your relationship with the government/local authorities. | | |  |
|  | Is the facility registered with a regulatory body? | Yes.................................................................................  No .................(Skip to Q66).......................................... | | 1  2 |
|  | If yes, which body? | Specify organization___________  Other (specify)___________________  Don’t Know………………………………………. | | 1  2  -999 |
|  | When was the last accreditation/authorization visit made to this facility by the government? | Less than 6 months ago………………………………………………  Between 6 and 12 months ago……………………………………  More than a year ago………………………………………………….  Never………………………………………………………………………….  Choose not to answer…………………………………………………  Don’t know………………………………………………………………… | | 1  2  3  4  -888  -999 |
|  | Does the Ministry of Health supervise your vaccination activities?  If yes, how often? | Yes ………..………………………………………………………………  No …………………………………………………………………………  Don’t know…………………………………………………………………  # Number of times per year | | 1  2  -999  _____ |
|  | Do you send monthly reports on vaccination conducted to district health authorities or headquarters?  . ASK TO SEE HOW DAILY VACCINATIONS ARE TALLIED, AND HOW MONTHLY FIGURES ARE RECORDED AND REPORTED TO VERIFY ANSWER..  Where do you send the reports? | Yes .……………………………………………………………………….  No ..…………………………………………………………..…………  Other (specify)_____________________________  Don’t know……………………………………………………………  District authorities………………………………………………..  Headquarters……………………………………………………….  Other (specify) …………………………………………………….  Don’t know………………………………………………………….. | | 1  2  -888  -999  3  4  -888  -999 |
|  | Does the government give you vaccines, injection equipment, registries, immunization cards..?  ASK TO SEE SUPPLY LEDGERS to verify answers. | Vaccines………………………………………………………..  Injection equipment (Syringes and safety boxes)………………………………………  Cold chain equipment…………………………………..  Other (specify)_____________  Don’t know……………………………. | | 1  2  3  -888  -999 |
|  | What is the source of your cold chain equipment (refrigerator, cold boxes, etc.)? | Government………………………………………………………..  Headquarters………………………………………………………  Purchase from distributor…………………………………..  Other (specify)____________________________  Don’t know………………………………………………………… | | 1  2  3  -888  -999 |
|  | Who provides training for your health workers on vaccination? | Organization.……………………………………………………………………….  National Immunization Program…………………………………………………………..…………  Other (specify)_____________________________  Don’t know…………………………………………………………… | | 1  2  -888  -999 |

# Section B – Vaccine Storage

The following are some questions on the storage of the vaccines:

|  |  | Availability | Code |
| --- | --- | --- | --- |
|  | Are routine vaccines stored at this health facility? | Yes  No  Other (specify)…………………………………….  Don’t know………………………………………….. | 1  2  -888  -999 |
|  | From where are the vaccines transported? | National Warehouse  Regional warehouse  District warehouse  Local Distributor  Other (specify)___________  Don’t know | 1  2  3  4  -888  -999 |
|  | Where are the vaccines stored in this facility?  (specify location) | EPI room  Child health room  Storage room  Other (specify)……………………………………..  Don’t know…………………………………………… | 1  2  3  -888  -999 |
|  | What kind of refrigerator or cold box is used to store vaccines at this facility? | Type of refrigerator _______________________________  Second refrigerator _______________________________  Cold box________________________________________  Other (specify)………………………………………………………………….  Don’t know……………………………………………………………………… | 1  2  3  -888  -999 |
|  | What is the source of energy for the refrigerator? | Electricity  Battery  Solar  Gas  Kerosene  Other (specify)  Don’t Know | 1  2  3  4  5  -888  -999 |
|  | Does the refrigerator have athermometer? | Yes  No  Don’t know……………………………………….. | 1  2  -999 |
|  | What is the source of funding for purchasing the cold chain equipment in this facility (refrigerator)? | Government  Purchased by headquarters  Purchased by other source (specify)__________________  Don’t know | 1  2  -888 -999 |
|  | What is the source of funding for running costs of cold chain equipment? | Government  Headquarters  Other source (specify)__________________  Don’t know | 1  2  -888  -999 |

Interviewer instructions: Check whether the following vaccines are in stock. For each vaccine, attempt to directly observe whether it is in stock. If this is not possible, ask whether it is in stock. If it is not in stock, ask whether they provide the vaccine but are out of stock.

|  |  | Availability | Code |
| --- | --- | --- | --- |
|  | BCG | In stock (observed)  In stock (reported)  Not available now but available at other times  Not stocked | 1  2  3  4 |
|  | DTP or Pentavalent | In stock (observed)  In stock (reported)  Not available now but available at other times  Not stocked | 1  2  3  4 |
|  | Measles | In stock (observed)  In stock (reported)  Not available now but available at other times  Not stocked | 1  2  3  4 |
|  | Measles-rubella (or MMR) | In stock (observed)  In stock (reported)  Not available now but available at other times  Not stocked | 1  2  3  4 |
|  | OPV | In stock (observed)  In stock (reported)  Not available now but available at other times  Not stocked | 1  2  3 4 |
|  | IPV | In stock (observed)  In stock (reported)  Not available now but available at other times  Not stocked | 1  2  3  4 |
|  | Pneumococcal (PCV) | In stock (observed)  In stock (reported)  Not available now but available at other times  Not stocked | 1  2  3  4 |
|  | Rotavirus | In stock (observed)  In stock (reported)  Not available now but available at other times  Not stocked | 1  2  3  4 |
|  | Tetanus Toxoid (TT)/Td | In stock (observed)  In stock (reported)  Not available now but available at other times  Not stocked | 1  2  3  4 |
|  | Influenza vaccine | In stock (observed)  In stock (reported)  Not available now but available at other times  Not stocked | 1  2  3  4 |
|  | HPV | In stock (observed)  In stock (reported)  Not available now but available at other times  Not stocked | 1  2  3  4 |
|  | Other vaccine (specify)________ | In stock (observed)  In stock (reported)  Not available now but available at other times  Not stocked | 1  2  3  4 |

|  | Interview finish time hh.mm  (Use 24 hour time) | . |
| --- | --- | --- |

# Section C – Prices of Vaccines and Injection Equipment

If vaccines are purchased (bulk) rather than donated, probe to see how much is being paid for these.

|  | **Question** | **Response** |  |
| --- | --- | --- | --- |
|  | Who purchases the vaccines for the facility (bulk)? | Facility Management………………………………………..  Organization Headquarters………………………………  Government……………………………………………………..  Other (specify)………………………………………………….  Don’t Know……………………………………………………… | 1  2  3  -888  -999 |
|  |  |  |  |
|  | **Vaccine** | **Presentation (e.g. 2 dose vial vs. 1 dose vial)** | **Cost per vial to the facility**  **(bulk price)** |
|  | BCG |  | ________ |
|  | DTP or Pentavalent |  | ________ |
|  | Measles  Measles-Rubella  Measles-Rubella-Mumps |  | ________  ________  ________ |
|  | OPV |  | ________ |
|  | IPV |  | ________ |
|  | Monovalent Hepatitis B |  | ________ |
|  | Pneumococcal |  | ________ |
|  | Rotavirus |  | ________ |
|  | Other (specify) HPV, Chickenpox, DT, YF |  | ________ |
|  | Other (specify) |  | _________ |
|  | **Injection Equipment** | **Type of injection equipment** | **Cost per unit** |
|  | Auto-disable Syringe, SAFETY BOXES |  |  |
|  | Reconstitution syringe |  |  |
|  | Other injection equipment (specify) |  |  |
|  | **Other (specify)** | **Unit** | **Cost per dose** |
|  | Other (specify) |  |  |
|  | Other (specify) |  |  |

Immunization Exit Interview

Form Number (Unique ID): ___________________

# Preliminary section: To be completed before beginning interview

| **S/N** | **Variable** | **Category** | **Code** |
| --- | --- | --- | --- |
|  | Interviewer name |  |  |
|  | Interviewer code |  |  |
|  | Client Code |  |  |
|  | Interview date | Day: □□ Month: □□ Year: □□□□ |  |
|  | District (or commune) name |  |  |
|  | Department or Region |  | 1 |
|  | Health Area |  | 1 |
|  | Is the facility located in a rural, urban or peri-urban area? | Rural……………………………………………………………………………………………………………  Peri-urban…………………………………………………………………………………………………  Urban…………………………………………………………………………………………………………. | 1  2  3 |
|  | Type of Facility | Private for profit clinic  Private for profit hospital  NGO clinic  NGO hospital  Public  Other (specify)_________________________________ | 1  2  3  4  5  8 |

##

| **READ TO CLIENT:** Hello, I am_____________. We are representing Abt Associates to find about the vaccination services here. We would like to ask you some questions about your experiences here today. | | | | | | | | | | | | | | | | | | | | | | | | | | | | | | | | | | | | | | | | | | | | | | | | | | | | | | | | | | | | | | | | | | | | | | | | | | | | | | | | | | | | | | | | | | | | | | | | | | | | | | | | | | | | | | | | | | | | | | | | | | | | | | | | | | | | | | | | | |  | | | | |  | | | | |  | | | | |  | | | | |  | | | | | |  | | | | | |  | | | | |  | | | |  | | | |  | | | | |  | | | | | |  |  |  |  |  |  |  |  |  |  |  |  |  |  |  |  |  |  |  |  |  |  |  |  |
| --- | --- | --- | --- | --- | --- | --- | --- | --- | --- | --- | --- | --- | --- | --- | --- | --- | --- | --- | --- | --- | --- | --- | --- | --- | --- | --- | --- | --- | --- | --- | --- | --- | --- | --- | --- | --- | --- | --- | --- | --- | --- | --- | --- | --- | --- | --- | --- | --- | --- | --- | --- | --- | --- | --- | --- | --- | --- | --- | --- | --- | --- | --- | --- | --- | --- | --- | --- | --- | --- | --- | --- | --- | --- | --- | --- | --- | --- | --- | --- | --- | --- | --- | --- | --- | --- | --- | --- | --- | --- | --- | --- | --- | --- | --- | --- | --- | --- | --- | --- | --- | --- | --- | --- | --- | --- | --- | --- | --- | --- | --- | --- | --- | --- | --- | --- | --- | --- | --- | --- | --- | --- | --- | --- | --- | --- | --- | --- | --- | --- | --- | --- | --- | --- | --- | --- | --- | --- | --- | --- | --- | --- | --- | --- | --- | --- | --- | --- | --- | --- | --- | --- | --- | --- | --- | --- | --- | --- | --- | --- | --- | --- | --- | --- | --- | --- | --- | --- | --- | --- | --- | --- | --- | --- | --- | --- | --- | --- | --- | --- | --- | --- | --- | --- | --- | --- | --- | --- | --- | --- | --- | --- | --- | --- | --- | --- | --- | --- | --- | --- | --- | --- | --- | --- | --- | --- | --- | --- | --- | --- | --- | --- | --- | --- | --- | --- | --- | --- |
| Please know that whether you decide to allow this interview or not is completely voluntary and will | | | | | | | | | | | | | | | | | | | | | | | | | | | | | | | | | | | | | | | | | | | | | | | | | | | | | | | | | | | | | | | | | | | | | | | | | | | | | | | | | | | | | | | | | | | | | | | | | | | | | | | | | | | | | | | | | | | | | | | | | | | | | | | | | | | | | | | | | | | | | | | | | | | | | | | | | | | | | | | | | | | | | | | | | | | | | | | | | | | | | | | | | | | | | | | | | |  |  |  |  |  |  |  |  |  |  |  |  |  |  |  |  |  |  |  |  |  |  |  |  |
| not affect services you receive during any future visit. You may refuse to answer any question, and | | | | | | | | | | | | | | | | | | | | | | | | | | | | | | | | | | | | | | | | | | | | | | | | | | | | | | | | | | | | | | | | | | | | | | | | | | | | | | | | | | | | | | | | | | | | | | | | | | | | | | | | | | | | | | | | | | | | | | | | | | | | | | | | | | | | | | | | | | | | | | | | | | | | | | | | | | | | | | | | | | | | | | | | | | | | | | | | | | | | | | | | | | | | | | | | | |  |  |  |  |  |  |  |  |  |  |  |  |  |  |  |  |  |  |  |  |  |  |  |  |
| you may stop the interview at any time. | | | | | | | | | | | | | | | | | | | | | | | | | | | | | | | | | | | | | | | | | | | | | | | | | | | | | | | | | | | | | | | | | | | | | |  | | | |  | | | | |  | | | |  | | | |  | | | | |  | | | | |  | | | |  | | | | |  | | |  | | | | |  | | | |  | | | | |  | | | | |  | | | | | |  | | | |  | | | | |  | | | | |  | | | | |  | | | | |  | | | | | |  | | | | | |  | | | | |  | | | |  | | | |  | | | | |  | | | | | |  |  |  |  |  |  |  |  |  |  |  |  |  |  |  |  |  |  |  |  |  |  |  |  |
|  |  | | | |  | | | |  | | |  | | |  | | |  | | | |  | | | |  | | |  | | | |  | | | |  | | |  | | | | |  | | | |  | | | | |  | | | |  | | |  | | | |  | | | | |  | | | |  | | | | |  | | | |  | | | |  | | | | |  | | | | |  | | | |  | | | | |  | | |  | | | | |  | | | |  | | | | |  | | | | |  | | | | | |  | | | |  | | | | |  | | | | |  | | | | |  | | | | |  | | | | | |  | | | | | |  | | | | |  | | | |  | | | |  | | | | |  | | | | | |  | | | | | | | | | | | | | | | | | | | | | | | |
| Information from this interview may be provided to researchers for analyses, but neither your name nor | | | | | | | | | | | | | | | | | | | | | | | | | | | | | | | | | | | | | | | | | | | | | | | | | | | | | | | | | | | | | | | | | | | | | | | | | | | | | | | | | | | | | | | | | | | | | | | | | | | | | | | | | | | | | | | | | | | | | | | | | | | | | | | | | | | | | | | | | | | | | | | | | | | | | | | | | | | | | | | | | | | | | | | | | | | | | | | | | | | | | | | | | | | | | | | | | |  |  |  |  |  |  |  |  |  |  |  |  |  |  |  |  |  |  |  |  |  |  |  |  |
| the date of services will be on any shared information, so your identity will remain completely | | | | | | | | | | | | | | | | | | | | | | | | | | | | | | | | | | | | | | | | | | | | | | | | | | | | | | | | | | | | | | | | | | | | | | | | | | | | | | | | | | | | | | | | | | | | | | | | | | | | | | | | | | | | | | | | | | | | | | | | | | | | | | | | | | | | | | | | | | | | | | | | | | | | | | | | | | | | | | | | | | | | | | | | | | | | | | | | | | | | | | | | | | | | | | | | | |  |  |  |  |  |  |  |  |  |  |  |  |  |  |  |  |  |  |  |  |  |  |  |  |
| confidential. | | | | | | | | | | | | | | | | | | | | | | | |  | | | |  | | |  | | | |  | | | | | |  | | | | |  | | | | |  | | | | |  | | | |  | | | |  | | | | |  | | | | | |  | | | | |  | | | | |  | | | | |  | | | | |  | | | | |  | | | | |  | | | | |  | | | | |  | | | | |  | | | | |  | | | | | |  | | | | | |  | | | | | | |  | | | | |  | | | | | |  | | | | |  | | | | | |  | | | | | | |  | | | | |  | | | | | |  | | | | |  | | | | | |  | | | |  | | | |  | | | |  | | | | |  | | |  | | |
|  | | | |  | | | |  | | | |  | | | |  | | | |  | | | |  | | | |  | | |  | | | |  | | | | | |  | | | | |  | | | | |  | | | | |  | | | |  | | | |  | | | | |  | | | | | |  | | | | |  | | | | |  | | | | |  | | | | |  | | | | |  | | | | |  | | | | |  | | | | |  | | | | |  | | | | |  | | | | | |  | | | | | |  | | | | | | |  | | | | |  | | | | | |  | | | | |  | | | | | |  | | | | | | |  | | | | |  | | | | | |  | | | | |  | | | | | |  | | | |  | | | |  | | | |  | | | | |  | | |  | | |
| Do you have any questions for me? Do I have your permission to continue with the interview? | | | | | | | | | | | | | | | | | | | | | | | | | | | | | | | | | | | | | | | | | | | | | | | | | | | | | | | | | | | | | | | | | | | | | | | | | | | | | | | | | | | | | | | | | | | | | | | | | | | | | | | | | | | | | | | | | | | | | | | | | | | | | | | | | | | | | | | | | | | | | | | | | | | | | | | | | | | | | | | | | | | | | | | | | | | | | | | | | | | | | | | | | | | | | | | | | | | | | | | | | | | | | | | | | | | | | | | | | |
|  | | |  | | | |  | | | |  | | |  | | | | |  | | | |  | | | |  | | | | |  | | | |  | | | | | |  | | | | |  | | | | |  | | | | |  | | | | |  | | | | |  | | | | |  | | | | |  | | | | |  | | | | | |  | | | | | |  | | | | |  | | | | |  | | | | | | |  | | | | |  | | | | |  | | | | |  | | | | | |  | | | |  | | | | | |  | | | | |  | | | | |  | | | | |  | | | | | |  | | | | | |  | | | | |  | | |  | | | | |  | | | | |  | | | | | |  | | | | |  | | |  | | | |  | | | |  |  | | | | | | |  |
|  | | |  | | | |  | | | |  | | |  | | | | |  | | | |  | | | |  | | | | |  | | | |  | | | | | |  | | | | |  | | | | |  | | | | |  | | | | |  | | | | |  | | | | |  | | | | |  | | | | |  | | | | | |  | | | | | |  | | | | |  | | | | |  | | | | | | |  | | | | |  | | | | |  | | | | | | | | |  | | | | | | | | | |  | | | | | | | | | | |  | | | | | | | | | | **2** | | | | | | | | | | **0** | | | | | | | | | | **1** | | | | | | | | | | |  | | | | | |  | | |  |  |  |  |  |  |  |  |  |  |  |  |  |  |  |  |  |
|  | | |  | | | |  | | | |  | | |  | | | | |  | | | |  | | | |  | | | | |  | | | |  | | | | | |  | | | | |  | | | | |  | | | | |  | | | | |  | | | | |  | | | | |  | | | | |  | | | | |  | | | | | |  | | | | | |  | | | | |  | | | | |  | | | | | | |  | | | | |  | | | | |  |  |  |  |  |  |  |  |  |  |  |  |  |  |  |  |  |  |  |  |  |  |  |  |  |  |  |  |  |  |  |  |  |  |  |  |  |  |  |  |  |  |  |  |  |  |  |  |  |  |  |  |  |  |  |  |  |  |  |  |  |  |  |  |  |  |  |  |  |  |  |  |  |  |  |  |  |  | | |  |  |  |  |  |  |  |  |  |  |  |  |  |  |  |  |  |
| Interviewer's signature | | | | | | | | | | | | | | | | | | | | | | | | | | | | | | | | | | | | | | |  | | | | |  | | | | | |  | | | | |  | | | |  | | | |  | | | | |  | | | | |  | | | | |  | | | | | |  | | | | |  | | | |  | | | | |  | | | | |  | | | | |  | | | | |  | | | | | | DAY | | | | | | | | | | | | | | | | | | | | | | MONTH | | | | | | | | | | | | | | | | | | | | | | | | | | YEAR | | | | | | | | | | | | | | | | | | | | | | | | | | | | | | | | | | |  | | | |  |  |  |  |  |  |  |  |  |  |  |  |
| (Indicates respondent's willingness to participate) | | | | | | | | | | | | | | | | | | | | | | | | | | | | | | | | | | | | | | | | | | | | | | | | | | | | | | | | | | | | | | | | | | | | | | | | | | | | | | | | | | | | | | | | | | | | | | | | | | | | | | |  | | | | |  | | | | |  | | | | | |  | | | | |  | | | | |  | | | | | |  | | | | | |  | | | | |  | | | | | | | |  | | | | |  | | | | | |  | | | | | | |  | | | |  | | | | | | | | |  | | | | |  | | | | | |  | | |  | | |  | | | |  | | | | |  | | |  | | | |
|  | |  | | | |  | | | |  | | |  | | | |  | | | |  | | | |  | | | | |  | | | |  | | | |  | | | | |  | | | | |  | | | | |  | | | |  | | | |  | | | | |  | | | | |  | | | | |  | | | | |  | | | | |  | | | | |  | | | | |  | | | | | |  | | | | |  | | | | |  | | | | |  | | | | |  | | | | |  | | | | | |  | | | | | |  | | | | | |  | | | | |  | | | | | |  | | | | | |  | | | | | |  | | | | | |  | | | | | |  | | | | | |  | | | | |  | | | | | |  | | |  | | | |  | | | |  | | | | |  | | |  | |

# Section A – Basic Questions

| **Number** | **Question** | | **Answer** | **Code** |
| --- | --- | --- | --- | --- |
|  | May I begin the interview now? | | Agrees…………………………………………………….  Client Refuses…………………………………………. | 1  2 |
| 1. 1 | Interview start time  (Use 24 hour time) | | hh.mm | . |
|  | Why did you come to the clinic today? | | Curative Care…………………………………………….  Well baby visit…………………………………………..  Vaccination……………………………………………….  Antenatal care…………………………………………..  Other(specify)________________ | 1  2  3  4  5 |
| 1. . | Did you or your child receive a vaccination at this facility today? | | Yes……………………………………………………(skip Q6)  No, …………………………. | 1  2 |
|  | If you did not receive a vaccination today, was it due to a stockout, lack of funds, or bad service? | | Stockout  Lack of funds  Bad service  Not applicable | 1  2  3  -999 |
|  | Who received the vaccination today?  IF THERE IS MORE THAN ONE CHILD, ask about the youngest one? | | Child…………………………………………………………..  Pregnant woman………………………………………..  Other(specify)________________________ | 1  2  8 |
|  | Do you have a vaccination card/book, or a vaccination card with you today? | | Yes…………………………………………………………….  No, card kept with facility………………………….  No card/book used…………………………………… | 1  2  3 |
| 1. . | Check the child vaccination card, or ANC card (or card for adolescent girl). Indicate whether there is any note or record of the client or child having received a vaccination today. | | Yes, 1 time…………………………………………………  Yes, 2 times………………………………………………  Yes, 3 or more times………………………………..  No record…………………………………………………. | 1  2  3  4 |
|  | What vaccination(s) were provided to the client today? | BCG…………………………………………………………..  Pentavalent………………………………………………  OPV………………………………………………………….  PCV………………………………………………………….  Rotavirus………………………………………………….  Measles……………………………………………………  Measles Rubella (or MMR)……………………….  IPV……………………………………………………………  TT or Td……………………………………..  Influenza………………………………………………….  Other (specify)…………………………………………  Don’t know…………………………………………… | | 1  2  3  4  5  6  7  8  9  10  88  -999 |
| 1. . | During this visit (or previous visits), did a provider discuss with you the possible side effects of the vaccination? | | Yes, this visit only……………………………………  Yes, this & previous visit…………………………  Yes, previous visit only……………………………  No…………………………………………………………..  Don’t know……………………………………………. | 1  2  3  4  8 |
|  | Please tell me any side effects of the vaccinations.  PROBE: ANY OTHER? | | Fever………………………………………………………  Other(specify)________________  Don’t know……………………………………………. | 1  2  8 |
|  | During this visit (or previous visits) has a provider told you if any additional vaccinations are required (for you or your child?) and when to come back for additional vaccinations | | Yes, this visit only…………………………………..  Yes, this & previous visit…………………………  Yes, previous visit only……………………………  No…………………………………………………………..  Don’t know……………………………………………. | 1  2  3  4  8 |
|  | Did the provider answer all the questions that you had about the vaccinations? | | Yes, this visit only…………………………………..  Yes, this & previous visit…………………………  Yes, previous visit only……………………………  No…………………………………………………………..  Don’t know……………………………………………. | 1  2  3  4  8 |
| Now I am going to ask you some questions about the services you received today. I would like to have your opinion about the services you received. | | | | |
|  | How long did you wait between the time you arrived at this facility and the time you were able to see a provider for the health services? | | Minutes__________________  Saw provider immediately------------------------  Don’t know.……………………………………………………. | 1  2  8 |
|  | Please tell me if you had any problems today:   1. Time you waited to see a provider…………………………………… 2. Ability to discuss problems or concerns…………………………………. 3. Ask for clarifications? 4. Amount of explanation you received……………………………… 5. Availability of vaccines at the facility ………………………………. 6. The hours of service at this facility…………………………………. 7. The number of days services are available 8. The cleanliness of the facility…………….. 9. How the staff treated you…………………. 10. Cost for services or treatment….. 11. PROBE ABOVE ANSWERS…. | | Major Minor No problem DK  1 2 3 8  1 2 3 8  1 2 3 8  1 2 3 8  1 2 3 8  1 2 3 8  1 2 3 8  1 2 3 8  1 2 3 8 |  |
|  | Are you a part of any prepayment plan (such as medical aid, insurance or similar program) or institutional arrangement that pays for some or all of the services you [or your child?] receive at this or any other facility? | | Yes……………………………………………………………..  No………………………………………………………………  Don’t Know………………………………………………… | 1  2  8 |
|  | Were you charged, or did you pay fees for any services you [or your child?] received or were provided today? | | Yes……………………………………………………………..  No……………………………………………………(skip Q 18)  Don’t Know………………………………………………… | 1  2  8 |
|  | Which services were you charged for today? | | Registration…………………………………………………  Consultation………………………………………………  Vaccination  Syringes……………………………………………………..  Other supplies (specify)_________________  Curative care………………………………………………  ANC…………………………………………………………….  Other(specify)_________________________  Don’t know…………………………………………………. | 1  2  3  4  5  6  7  8  -888 |
|  | What is the amount you paid? | | Registration_________________________  Consultation_________________________  Vaccination____________________________  Syringes_____________________________  Other supplies (specify)________________  Curative care_________________________  ANC________________________________  Other(specify)________________________  Don’t know__________________________ |  |
|  | Is this the closest health facility to your home? (If yes, skip to 20) | | Yes…………………………………………………………….  No…………………………………………………………….  Don’t know……………………………………………… | 1  2  8 |
|  | What was the main reason you did not go to the facility nearest to your home? | | Inconvenient operating hours………………..  Bad reputation………………………………………..  Don’t like health workers…………………………  No medicine, vaccines, or supplies..…………………………  More expensive……………………………………….  Was referred……………………………………………  Other (specify)………………………………………….  Don’t know…………………………………………….. | 1  2  3  4  5  6  7  8 |
|  | In general, IF YOU or your child RECEIVED VACCINATION TODAY, which of the following statements describes your opinion of the vaccination services you or your child received today: PROBE: | | I am very satisfied with the services I received in facility………………………………………………………  I am more or less satisfied with the services I received………………………………………………………  I am not satisfied with the services I received | 1  2  3 |
| Now I am going to ask you some questions about yourself. | | | | |
|  | How old were you at your last birthday? | | Age in years______________________  Don’t know………………………………………………. |  |
|  | What is the highest level of school you attended? | | None  Some Primary  Completed primary  Post-primary/Vocational  Some Secondary  Completed secondary  College or greater | 1  2  3  4  5 |
|  | Do you have any questions or comments to make about the vaccination services in this facility? | | ____________________________ |  |
|  | Record the time the interview ended | | hh.mm |  |
| Thank you very much for taking the time to answer my questions. Your responses will be kept confidential. Have a nice day! | | | | |
| Interviewer’s comments: | | | | |
